# Assay Challenges for Emerging Infectious Diseases: The Zika Experience

**DOI:** 10.3390/vaccines6040070

**Published:** 2018-10-02

**Authors:** Christine C. Roberts, Joel N. Maslow

**Affiliations:** GeneOne Life Science, Inc., Seoul 60606, Korea; jmaslow@geneonels-us.com

**Keywords:** emerging infectious disease, vaccines, immune response, virus detection, zika virus, flaviviruses, clinical trials

## Abstract

From the perspective of vaccine development, it is imperative to accurately diagnose target infections in order to exclude subjects with prior exposure from evaluations of vaccine effectiveness, to track incident infection during the course of a clinical trial and to differentiate immune reactions due to natural infections from responses that are vaccine related. When vaccine development is accelerated to a rapid pace in response to emerging infectious disease threats, the challenges to develop such diagnostic tools is even greater. This was observed through the recent expansion of Zika virus infections into the Western Hemisphere in 2014–2017. When initial Zika vaccine clinical trials were being designed and launched in response to the outbreak, there were no standardized sets of viral and immunological assays, and no approved diagnostic tests for Zika virus infection. The diagnosis of Zika virus infection is still an area of active research and development on many fronts. Here we review emerging infectious disease vaccine clinical assay development and trial execution with a special focus on the state of Zika virus clinical assays and diagnostics.

## 1. Introduction

The declaration by the World Health Organization (WHO) that Zika is a public health emergency of international concern in February 2016 led to a global effort to support vaccine development and control the spread of Zika virus (ZIKV). Our collaborative DNA vaccine consortium focused and accelerated pre-clinical, manufacturing and early clinical development efforts to bring forward the first Zika vaccine, GLS-5700, into human clinical trials [1,2,3]. At the outset, it was clear that gaps would need to be filled as the public health and science communities learned and shared new information on Zika. One of the clear gaps affecting both public health efforts and vaccine development programs was a lack of standardized reagents and methods to test for evidence of current or prior Zika infection.

The need for fast and accurate diagnostic tests of infection in an outbreak situation is obvious: identify the source or epicenter so that appropriate healthcare measures can be quickly instituted. Expanding that concept to the public health scale and attaining accurate infectious disease diagnoses allows for better understanding of the course and severity of an outbreak and aids decision-making for population-level countermeasure implementation. The clinical assays with which the immune response and pathogen presence are measured in vaccine trials become part of the basis for licensure for all vaccine products [4]. Because vaccines are tested in healthy populations through all phases of clinical development for immune response and/or pathogen presence whereas drugs/biologics (post-Phase I) are most often tested in a population with specific disease to demonstrate improvement, the selected methods to measure vaccine responses and endpoints are of the utmost importance. The identification of an immune correlate of protection for each vaccine is highly desirable, though not always attainable [5]. Here we will use the recent experience with the ZIKV outbreak and ensuing public health countermeasures for containment and vaccine development as an example of challenges faced during emerging infectious disease emergencies.

## 2. Zika Virus Background

Zika virus was discovered in 1947 during a survey to map the extent of yellow fever in the Entebbe region of Uganda. The virus was cultured from the serum of a sentinel macaque placed in the Zika forest that developed fever but was otherwise well. Initially restricted to equatorial regions of Africa and Asia, ZIKV started to spread eastward across the Pacific Ocean with an outbreak on Yap Island, Micronesia in 2007 [6], in French Polynesia in 2014 [7], and reaching Brazil in late 2014 or early 2015 [8].

ZIKV infection typically causes a self-limited illness that is minimally symptomatic for most individuals. Zika infection presents similarly to dengue or chikungunya with fever in most, rash, malaise, myalgia, conjunctivitis and retro-orbital pain but may present with few, if any, discernable symptoms [9]. However, documentation in Brazil of severe neurologic complications associated with ZIKV infection beginning around October 2015 raised worldwide alert. The most publicized and dramatic complications are those that occur during fetal development. They include microcephaly, intra-uterine growth retardation, cerebral calcifications, ocular calcifications and other ocular abnormalities—with an attack rate estimated at 1–2% of women who become infected during pregnancy [10]. ZIKV can also directly infect the placenta and can result in spontaneous miscarriage with an unknown prevalence [11,12,13,14]. In adults, the most common complication of ZIKV infection is Guillain-Barré syndrome occurring at an estimated rate of 1 in 5000 cases [10]. Zika has also resulted in deaths among adults with and without other complicating factors [15,16].

There are no approved therapies or vaccines for ZIKV infection. There have been a number of vaccines developed for other flavivirus infections. Live virus vaccines utilizing chimeric viral constructs have been approved for use to prevent yellow fever and dengue. DNA vaccines have been developed and published for dengue [17,18], West Nile virus [18,19,20,21,22], and Japanese encephalitis virus [21]. Notably, DNA vaccines targeting West Nile virus [19,20] and dengue virus [17] have been tested as part of Phase I clinical trials without vaccine associated toxicity. Currently, as recently reviewed elsewhere, a number of vaccine modalities targeting ZIKV have reached early stage clinical trials and even more are in preclinical development [23,24,25].

ZIKV is spread primarily through *Aedes* species mosquitoes, mainly *Aedes aegypti*, but can be carried by other mosquito species [26] and does not appear to be transmission competent except for *Aedes* species [26,27,28,29,30,31]. *Aedes* mosquitoes also transmit other arboviruses such as dengue and chikungunya [30,32]. In fact, these infections are co-endemic in most regions and may cause concurrent infections [33,34,35,36]. Alternative nonmosquito-borne routes of ZIKV spread include: blood transfusions, breast milk [37], sexual transmission [38,39,40], and may include urine and saliva [41,42,43,44]. The additional potential routes of ZIKV transmission have increased the need for definitive monitoring and a variety of surveillance strategies to prevent disease spread [45,46,47,48,49].

## 3. Zika Diagnostics and Assays for Vaccine Development

In an outbreak situation, such as with Zika, it is important to have the ability to quickly develop both diagnostic kits for public health purposes and vaccine clinical assays to support pre-clinical studies and early stage clinical trials. Both were largely unavailable on a commercial scale or for widespread use at the outset of the Zika outbreak, though development ensued at a rapid pace upon the declaration of a worldwide public health emergency. Because there is significant homology between ZIKV and other cocirculating flaviviruses, detection and diagnosis has had the extra challenge of avoiding cross-reactivity without sacrificing sensitivity. While the avoidance of cross-reactivity is more easily engineered into molecular tests of virus RNA because primers or probes can be designed to be virus-, antigen- and serotype-specific [41,50,51,52,53,54,55,56,57], it is not as easily achieved for immunoassays [58,59,60,61,62,63].

### 3.1. Zika Diagnostics 

Diagnostic assays are often the same style tests as those used in vaccine development, but are for the intended purpose of identifying the source of a patient’s illness to enable the initiation of appropriate treatments by healthcare professionals. The need for sensitivity and specificity as it relates to clinical disease identification for patient treatment is of the utmost concern. Early in the Zika outbreak, the US Centers for Disease Control and some academic laboratories studying flaviviruses had assays developed for ZIKV that generally supported their own research interests [64,65,66,67,68,69,70]. Making sufficient quantities of these tests available for public health use while ensuring consistency and quality was a significant challenge. Additionally, cross-reactivity in a number of immunological assays and the short time frame in which viremia can be detected in bodily fluids necessitated the institution of an algorithm to confirm ZIKV infection that was based on a combination of risk factors, clinical symptoms and diagnostic test results [71]. The Centers for Disease Control (CDC) issued testing guidance for healthcare providers using algorithms that included use of molecular testing for pregnant or non-pregnant and symptomatic or non-symptomatic individuals, the types of specimens and timing of collection of specimens that would provide the most reliable results [72]. In addition, guidance algorithms were provided for the use and interpretation of ZIKV IgM assays to indicate recent exposure with or without accompanying molecular test results [72]. 

In July of 2016, United States (US) Health and Human Services (HHS) sponsored a HHS Summit to Accelerate Zika Diagnostics Development, recognizing the important need. At that point in time, very few tests had gained Emergency Use Authorization (EUA) from the Food and Drug Administration (FDA). The five serological Zika diagnostic kits currently authorized by the US FDA are shown in Table 1 and fourteen viral diagnostic kits in Table 2 [73]. At the FDA Medical Countermeasures webpage, links can also be found for each EUA approved molecular assay’s key [74] and performance [75] characteristics including instruments approved for use and limits of detection. Although no Zika diagnostic kit, serological or viral, has been fully approved by the FDA to date, the FDA has worked collaboratively with developers to accelerate both EUA approvals and the transition to formal full approvals of Zika diagnostic kits using standard review timelines [76]. A number of studies have evaluated the various EUA tests both for independent sensitivity and specificity [50,52,54,56,60,61,70,77] assessments of assay performance and as part of comparative studies [47,78,79,80,81] to determine those best to use for the diagnostic or epidemiological surveillance need. 

A large number of companies have also developed tests that remain classified as “Research Use Only” (RUO) for which emergency authorizations have not been granted. Other authorizations for emergency use have been granted to different diagnostic kits by other agencies, such as WHO’s Emergency Use Assessment and Listing procedures (EUALs) [82]. While many diagnostic kits use methods similar or identical to those that may be used to support a vaccine clinical trial, often they are focused more toward the support of a clinical patient diagnosis and may not be sensitive enough for use to support vaccine development.

### 3.2. Zika Vaccine Clinical Assays

Often there is not enough data at the outset of a vaccine program, but especially in the case of emerging infectious diseases, to understand what assays will be most useful or informative or will perhaps even provide a correlate of protection for the vaccine into the future. A few general assumptions can be made at the outset about which types of assays will be needed to detect vaccine-induced humoral and cellular immune responses and these will evolve over time. The typical go-to methods used for vaccine clinical assays are: antibody binding—enzyme-linked immunosorbent assays (ELISA); functional—virus neutralization or bactericidal; cellular—interferon gamma enzyme-linked immunospot assay (IFNγ-ELISpot) using the target antigen or antigen-derived peptide pools; and molecular or culture methods to detect the pathogen. The technologies used to develop and run these assays have improved over the years to allow for higher throughput, multiplexing, reduced sample volumes, and automation. Often, more tests of a larger variety are done early in a program and are then whittled down based on the usefulness of the data generated so that just the relevant few remain to support large trials and licensure [4].

When the first ZIKV vaccine trials commenced in late summer of 2016, even though a few Zika diagnostics had achieved EUA status, none were commercially available and were initially restricted to public health use only. Challenges to vaccine development centered around the determination of prior ZIKV exposure and immunity and determination of incident infection among study participants. Each of the serological assays listed in Table 1 detect IgM reflective of recent infection, however many, such as the MAC-ELISA and the InBios assay, will detect IgM against the ZIKV envelope which is the target antigen for many vaccines. IgG-based assays have not yet been validated primarily due to cross-reactivity between ZIKV and other flaviviruses, principally dengue. IgM immunoassays targeted to the NS1 antigen are generally more specific than those directed to the viral envelope [47]. In September 2016, we initiated the first clinical trial of the GLS-5700 ZIKV DNA vaccine in an endemic region, Puerto Rico, an area also known to have high rates of exposure to dengue.

Because no widely accepted standardized assays nor any international reference standards or reagents existed at the time of trial initiation, individual vaccine projects needed to rely on internally developed clinical assays to understand prior exposure and vaccine related immune responses. 

Similar to patient diagnostic tests for ZIKV and as mentioned above, there was no accepted “gold standard” for any of the immunoassays one might choose to develop or use in a vaccine program. The extensive experience of our collaborative DNA vaccine team allowed us to develop ZIKV-specific tests such as ELISA, virus microneutralization and ELISpot around the development and pre-clinical testing of our ZIKV plasmid DNA vaccine constructs [1,2,83,84]. These assays performed consistently on a pre-clinical scale and we were able to quickly expand their use to support our two Phase 1 GLS-5700 vaccine clinical trials. A concern always remains, however, that the lack of highly-characterized reagents and controls in early versions of vaccine clinical assays will result in difficulties in the maintenance of the assays through its full life cycle. Sourcing, batch-to-batch variability and overall quality of critical reagents, standards and controls can become an issue over time. Lack of standardization across the scientific field can be confounding in that interpretation of results from multiple “home brew” assays across labs are not directly comparable in the absence of an accepted international standard or a proficiency panel of samples [4]. 

The main methodologies used to detect incident ZIKV infection are currently molecular-based, mainly reverse transcriptase polymerase chain reaction (RT-PCR) [36,41,43,50,52,54,56,70,85] or varieties thereof [55,86,87], since culture methods can be both difficult and laborious [88,89,90]. Those viral detection systems with EUA approval are shown in Table 2. In clinical trials, identification of newly infected subjects over the treatment and follow-up periods are necessary to determine vaccine efficacy. As discussed earlier, for most individuals ZIKV infection is minimally symptomatic such that few present to clinical care. ZIKV is detectable in serum by RT-PCR for only a short interval following the onset of symptoms, typically for only seven days to a maximum of 14 days [42,43], though longer periods of RT-PCR detection (up to 53 days) have been observed in serum of pregnant women [53], which may contribute to the incidence of ZIKV-related birth defects. ZIKV is excreted into the urine for approximately four weeks in most individuals, though this observation was not documented until over a year into the epidemic [42,43,91,92]. Because of this, RT-PCR-based diagnosis of incident infections in a vaccine clinical trial would require very frequent sampling. Other sample types have been evaluated for RT-PCR detection including saliva, whole blood, plasma, brain tissue, amniotic fluid and vaginal secretions [41,43,54,90,93,94]. 

A key concern from developers of both diagnostics and of vaccines or therapeutics for ZIKV highlighted at the HHS Summit for Diagnostics in July 2016 was the lack of well-characterized human ZIKV specimens which groups could use to fully understand the performance characteristics of the assays being developed and, eventually, work toward some standardization across the field. The WHO has initiated in, July 2016, a collaborative study effort for the development of Nucleic Acid Testing International Standard for Zika [95]. Additionally, in July 2017, a plasma sample panel became available through the US FDA for use in evaluating ZIKV immunoassay performance [96]. 

Reagents for newly emergent infectious diseases like ZIKV were not readily available from commercial vendors that had consistent production methods and quality controls in place, thus many reagents were not well characterized early on in the development of ZIKV clinical assays. Because validated assays supporting vaccine efficacy endpoints need to support clinical and regulatory expectations over the life of the product, it is imperative that a line-of-sight is maintained such that reliable and qualified materials in appropriate quantity are available for resolving issues that arise. Assays typically require some troubleshooting over time, changes to reagent lots or instruments, potential for multiplexing, platform changes to increase testing throughput, or a desire to bridge to a new technology [4]. The development, qualification, validation and maintenance of vaccine clinical assays should be done in close consultation with biostatisticians and bench scientists to ensure the optimal assay design, control parameters and performance characteristics for the needs of the vaccine program from beginning to end.

It should be noted that vaccine assay quality is highly dependent upon clinical study execution from collection through final data reporting. Sample collection & handling are critical to the quality of the specimen and its ability to be used in an assay. Implementing methods to assure the following are keys to successful vaccine clinical trials: proper sample storage and shipping conditions, processing and aliquotting with methods for contamination control, proper training of site and clinical research organization lab personnel, chain of custody verification of samples from collection to final valid test result, quality control checks and good data management.

In the execution of vaccine clinical trials, there is the need for testing methodology to be reliable, reproducible (accuracy, specificity, robustness), and occasionally to provide rapid diagnosis (on-site or point-of-care testing, if needed), which contributes to patient care as well as to the understanding of vaccine efficacy or disease epidemiology. 

## 4. Summary and Emerging Infectious Disease (EID) Future Preparedness

The diagnostic assay development response to ZIKV was quite rapid with the EUA approval of 14 different molecular detection assays and five serological assays in roughly 18 months’ time (Table 1 and Table 2) and the initiation of efforts to build international reference standards for both assay types. However, at the time of writing, there are still no fully approved diagnostics, no established “gold standard” detection methods nor any fully characterized and accepted international reference standards for ZIKV. While our understanding of the immune response to ZIKV has greatly expanded since the start of the most recent outbreak and leverages the years of vaccine research for other flaviviruses, such as dengue, there is still no established immune correlate of protection. Other flavivirus vaccines have established immune correlates that are based on neutralization titers and it has been assumed that ZIKV will, as well [97]. Post-vaccination serum from participants enrolled in our group’s GLS-5700 Zika DNA vaccine phase 1 trial protected 92% of interferon α/β receptor knockout mice (IFNAR) in a lethal-challenge model of ZIKV infection, however this protection was not dependent upon neutralizing antibody titers. Other vaccines in clinical development have achieved neutralizing antibody titers in humans which were similar to the titers conferring protection in pre-clinical studies of the vaccines [98,99]. Serum from participants in a phase 1 clinical study receiving a purified inactivated ZIKV vaccine was also able to protect mice in a passive transfer mouse model [98]. The development and validation of sensitive, specific and standardized ZIKV immunoassays to better characterize the immune response to vaccination are necessary to work toward the establishment of an immune correlate of protection in humans. 

Efforts are being made through the work of the Coalition for Epidemic Preparedness Innovations (CEPI) and others to ensure that rapid response mechanisms are in place to address emerging infectious diseases. Well-seasoned development teams have accepted the challenges and funding to support building the vaccine design, manufacture and clinical assessment infrastructure needed to save lives in outbreak situations. Scientific and quality principles must still apply although speed may be required when developing vaccine or diagnostic assays during an outbreak of a new emerging infectious disease. EID public health and countermeasure programs often have unique challenges for diagnostic and vaccine clinical assay development purposes including:

1. Incomplete understanding of biology or epidemiology for appropriate target selection.
2. Lack of available reagent sources; inconsistency in quantity and/or quality of those available.
3. Difficulty in obtaining relevant human sample panels for the evaluation of test methods.
4. Challenges to produce clear line of sight sourcing and quality from early vaccine development through licensure.

Although the speed at which the diagnostic or vaccine development field needs to move will be dependent upon the urgency of the emerging infectious pathogen and its impact on human life, biological assay standardization is a critical component and requires three separate activities:

- Development of relevant, sensitive, specific and preferably quantitative biological assays.
- Use of biostatistics to analyze assay performance data from development to validation to life cycle performance management.
- Ability to prepare stable and reproducible results over time to support diagnostic or vaccine program needs.

If assay performance consistency and quality is not demonstrated, the validity of clinical study results may be questioned.

Assay standardization for any newly emergent infectious disease will be challenging and will: (i) take time to develop, collect and characterize quality reagents, (ii) to achieve sufficient sources of confirmed positive samples for establishment of serological standards, and (iii) to confirm new molecular standards, though this is more straightforward for molecular assays than serology. Any efforts to prepare reagents, collect characterized samples, develop research materials and international standards in advance for EIDs for whom alerts have already been raised will leave the field better suited to respond in case of emergency.

## Figures and Tables

**Table 1:** US FDA Emergency Use Authorized Zika Serological Diagnostic Tests.

Zika Diagnostic Test	Manufacturer	Date of EUA	Target	Sample Types ^1^
Zika MAC-ELISA	CDC	26 February 2016	Inactivated Cell Culture or Zika VLP Antigen	S, CSF
ZIKV Detect 2.0 IgM Capture ELISA	InBios International	17 August 2016	E	S
LIAISON® XL Zika Capture IgM Assay	DiaSorin Incorporated	5 April 2017	NS1	S
ADVIA Centaur Zika	Siemens Healthcare Diagnostics	18 September 2017	NS1	S, P
DPP Zika IgM Assay System	Chembio Diagnostic Systems	27 September 2017	NS1	F, S, P, WB

^1^ F = Finger-stick Blood, CSF = Cerebrospinal Fluid, P = EDTA Plasma, S = Serum, WB = EDTA Whole Blood.

**Table 2:** US FDA Emergency Use Authorized Zika Viral Diagnostic Tests.

Zika Diagnostic Test	Manufacturer	Date of EUA	Target ^1^	Sample Types ^2^
Trioplex Real-time RT-PCR Assay	CDC	17 March 2016	E	S, WB, CSF, AF, U
Zika Virus RNA Qualitative Real-Time RT-PCR	Quest Diagnostics	28 April 2016	E, M	S, U
RealStar® Zika Virus RT-PCR Kit U.S.	altona Diagnostics	13 May 2016	-	S, EP, U
Aptima® Zika Virus Assay	Hologic	17 June 2016	NS2, NS4/5	S, P, U*, WB*
Zika Virus Real-time RT-PCR Test	Viracor Eurofins	19 July 2016	-	S, P, U
VERSANT® Zika RNA 1.0 Assay (kPCR) Kit	Siemens Healthcare Diagnostics	29 July 2016	-	S, EP, U
xMAP® MultiFLEX™ Zika RNA Assay	Luminex Corporation	4 August 2016	-	S, P, U
Sentosa® SA ZIKV RT-PCR Test	Vela Diagnostics USA	23 September 2016	NS4	S, EP, U
Zika Virus Detection by RT-PCR Test	ARUP Laboratories	28 September 2016	-	S, EP, U
Abbott RealTime Zika	Abbott Molecular	21 November 2016	prM, NS3	S, EP, WB, U
Zika ELITe MGB® Kit U.S.	ELITech Group Inc. Molecular Diagnostics	9 December 2016	NS3	S, EP
Gene-RADAR® Zika Virus Test	Nanobiosym Diagnostics	20 March 2017	-	S
TaqPath Zika Virus Kit	Thermo Fisher Scientific	2 August 2017	-	S, U
CII-ArboViroPlex rRT-PCR	Columbia University	11 August 2017	3’ UTR	S, U

^1^ E = Envelope, prM = preMembrane, M = Membrane, NS = Non-structural Protein, UTR = Untranslated Region, (-) = Proprietary. ^2^ AF = Amniotic Fluid, CSF = Cerebrospinal Fluid, EP = EDTA Plasma, P = Plasma, S = Serum, U = Urine, WB = Whole Blood (* = processed).
